# New Roles of Chk1 in Spindle Formation and Genome Integrity

**DOI:** 10.3390/biology15141105

**Published:** 2026-07-09

**Authors:** Sofia Balafouti, George Zachos, Eleni Petsalaki

**Affiliations:** Department of Biology, University of Crete, Vassilika Vouton, 70013 Heraklion, Greece; molgrad417@edu.biology.uoc.gr (S.B.); gzachos@uoc.gr (G.Z.)

**Keywords:** mitosis, microtubules, mitotic spindle, centrosomes, Chk1, β-tubulin, genome integrity

## Abstract

During mitosis sister chromatids are accurately segregated into two daughter cells through a microtubule-based structure known as the mitotic spindle. Errors in mitotic spindle formation have been associated with human diseases and tumorigenesis. Chk1 has well established roles in DNA damage response and replication checkpoints. Recently, new roles have emerged in the onset of mitosis, chromosome segregation and cytokinesis. In this review, we recapitulate the known roles of Chk1 in cell division and highlight new roles of Chk1 in mitotic spindle assembly and its contribution to genome stability. Specifically, it was recently reported that, during the first stages of mitosis, ATRIP and its interacting partners, ATR and TopBP1, are recruited to centrosomes, where they activate Chk1. Then, Chk1 phosphorylates β-tubulin at Threonine 285 (T285), promoting microtubule nucleation and mitotic spindle formation. β-tubulin phosphorylation by Chk1 is required for proper mitotic progression, cytokinesis with equal-sized daughter cells and cell proliferation. This novel ATR-Chk1 signaling pathway reinforces the crosstalk between the cell cycle regulation and DNA damage response.

## Simple Summary

Chk1, a Serine Threonine kinase, is a key regulator of DNA damage response. In the presence of replication stress or DNA damage, ATR phosphorylates and activates Chk1, promoting cell cycle arrest, whereas Chk1 is also implicated in unperturbed mitosis. In the absence of DNA damage, Chk1 ensures proper mitosis onset and monitors defects in kinetochore–microtubule attachments. This review summarizes the roles of Chk1 in cell division and demonstrates its emerging functions in mitotic spindle formation and genome integrity.

## 1. Introduction

Mitosis is a crucial stage of the cell cycle in which duplicated chromosomes are accurately segregated into two daughter cells and this segregation is mediated by a microtubule-based structure called the mitotic spindle [1,2,3]. Mitotic spindle assembly is a principal key of cell proliferation that ensures the maintenance of genome integrity in eukaryotic cells. Defects in mitotic spindle formation can lead to spindle malorientation, chromosome segregation errors and unequal-sized daughter cells, thereby increasing chromosomal instability and contributing to cancer development [4,5,6]. However, the mechanisms underlying mitotic spindle formation remain incompletely understood.

### 1.1. Microtubule Structure

Microtubules (MTs) are the major components of the mitotic spindle and are characterized by alternating phases of GTP-dependent polymerization and depolymerization [7]. Dynamic α/β-tubulin heterodimers associate head to tail to form linear protofilaments. Every tubulin monomer is composed of 12 α-helices (H1–H12) and 10 β-strands (S1–S10) that are associated through loops [8,9]. A total of 13 protofilaments interact laterally to form hollow cylindrical MTs exhibiting a “minus” (α-tubulin) end and a “plus” (β-tubulin) end [10,11].

### 1.2. Mitotic Spindle Assembly

**Figure 1:**
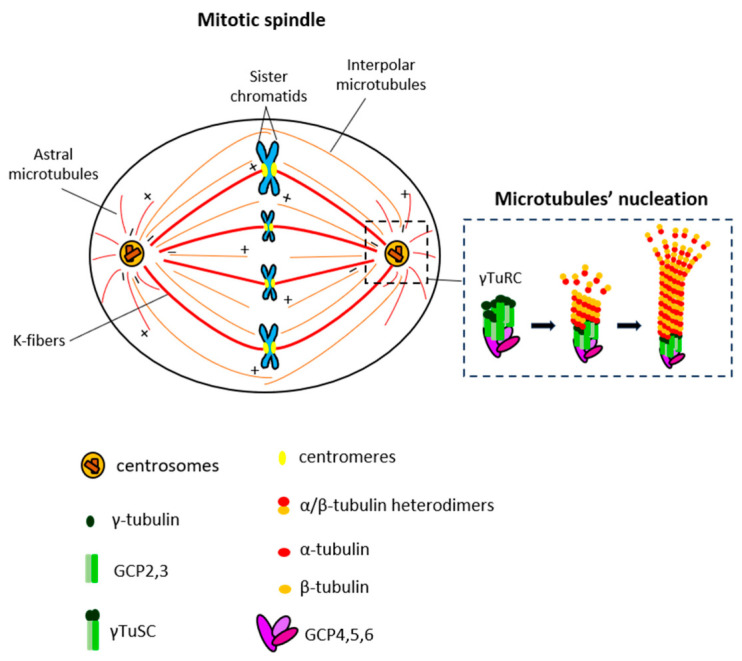
Mitotic spindle assembly. At the beginning of mitosis, γ-tubulin accumulates at centrosomes and forms a complex with GCP2 and GCP3, known as the γ-tubulin small complex (γTuSC). Multiple γTuSCs together with GCP4, GCP5 and GCP6 form the γ-tubulin ring complex (γTuRC), which acts as a template for microtubule nucleation. α/β-tubulin heterodimers are added to the plus (+) end of elongating microtubules, promoting their polymerization. The bipolar spindle microtubules, which extend from the centrosomes towards the cell cortex and spindle midzone, are categorized into astral microtubules, interpolar microtubules and kinetochore microtubules (K-fibers).

In animal cells, bipolar spindle microtubules are divided into three categories: kinetochore, astral and interpolar MTs. First, kinetochore microtubules extend from the spindle poles toward the chromosomes until their plus ends become attached to the kinetochores of sister chromatids [1]. Microtubules employ a search and capture mechanism by polymerizing and depolymerizing and exploring the inner space of the cell until their plus (+) ends attach to sister kinetochores. These attachments are called spindle kinetochore microtubule attachments [12,13,14]. Approximately 20–30 kinetochore microtubules bundle together to form kinetochore fibers (K-fibers) [15]. When all kinetochores are attached with spindle microtubules emanating from opposite poles and are aligned in the metaphase plate, the cell is ready to move to the next phase called anaphase [16,17,18]. Second, astral microtubules extend from the poles towards the cell cortex and are implicated in spindle positioning, whereas interpolar microtubules extend from the opposite poles towards the spindle midzone where they overlap and contribute to spindle stability [1,15] (Figure 1). In anaphase, kinetochore microtubules become shorter and move to the spindle poles leading to chromosome segregation [19,20,21]. The last phase of mitosis, which is called cytokinesis, divides the cell into two identical daughter cells [22].

To ensure effective interaction between kinetochores and the “+” ends of spindle microtubules, centromere-localized proteins, such as CENP-A recruit subunits of the Constitutive Centromere-Associated Network (CCAN) [23]. In turn, CCAN connects to the KNL1/Mis12 complex/Ndc80 complex (KMN) network, which binds directly to microtubules [24,25]. Proteins of the CENP family play important roles in the mitotic regulation. CENP-A localizes to the centromeres and contributes to the recruitment of kinetochore proteins [23,26]. CENP-E and CENP-F localize to the outer kinetochores in a BUB-dependent manner and are required for initial microtubule–kinetochore attachment [27]. CENP-B localizes to the inner centromere and it has DNA-binding ability [23]. CENP-C is a subunit of CCAN and localizes to the inner kinetochores [28,29]. CENP-C binds both chromatin and kinetochore components, thereby linking the centromeric chromatin to the outer kinetochore and mediating microtubule attachment [30,31,32].

Bipolar attachment of replicated chromosomes to K-fibers is essential for faithful chromosome segregation during mitosis [33,34]. A lack of bipolar microtubule–kinetochore attachment activates the spindle assembly checkpoint (SAC) (also known as the mitotic spindle checkpoint), which prevents anaphase onset [35,36]. The SAC signaling inhibits the activation of anaphase-promoting complex/cyclosome (APC/C), an E3 ubiquitin ligase, that is required for chromosome segregation and mitotic exit [35,37,38,39]. In the presence of unattached kinetochores, SAC components such as Bub1, BubR1, Bub3, Mps1, Mad1, Mad2 and Cdc20 are recruited to kinetochores to block the activity of APC/C^Cdc20^ complex [40,41,42,43,44]. Mad2, BubR1, Bub3 and Cdc20 are components of a tetrameric complex called the mitotic checkpoint complex (MCC) [45]. Cdc20 has a dual role in SAC. During mitosis, APC/C binds to its co-activator, Cdc20, forming the APC/C^Cdc20^ complex that ubiquitinates downstream effectors to promote mitosis exit [46,47]. However, during SAC signaling, Cdc20, as a subunit of MCC, binds to APC/C^Cdc20^ complex and inhibits its activity [48]. Dysfunction of the SAC leads to premature segregation of sister chromatids, lagging chromosomes, chromosome bridges and formation of micronuclei, which are associated with increased chromosomal instability and cancer [49,50].

In eukaryotic cells, the organizing centers of microtubules are called centrosomes and are formed around the centrioles [51]. During interphase, γ-tubulin localizes at centrosomes in low levels. But, at the onset of mitosis, γ-tubulin accumulates at centrosomes rapidly, where it forms a complex with γ-tubulin complex proteins (GCPs) [1,52,53]. Two copies of γ-tubulin and one of GCP2 and GCP3 form the γ-tubulin small complex (γTuSC), which has a limited capacity for microtubule nucleation. Numerous copies of γTuSC together with GCP4, GCP5 and GCP6 form the γ-tubulin ring complex (γTuRC), which acts as a template for the positioning of α/β-tubulin heterodimers and promote microtubule polymerization [53] (Figure 1). Aurora A and polo-like kinase 1 (Plk1) recruit γ-tubulin to the centrosome, promoting centrosome maturation and microtubule nucleation [54,55]. At centrosomes, DNA damage response (DDR) proteins, checkpoint kinase 2 (Chk2) and breast cancer type 1 susceptibility protein (BRCA1), regulate Aurora A catalytic activity, whereas MRN (Mre11-Rad50-Nbs1) protein complex supports Plk1 activity to promote mitotic spindle formation (reviewed in [56]). Alterations at the localization and expression pattern of γ-tubulin have been observed in many tumors, including breast and lung cancer [57,58,59]. In vertebrates, additional centrosome-independent mechanisms of the spindle assembly have been demonstrated, which increase the microtubule density around the kinetochores, including microtubule nucleation from chromosomes, kinetochores or within the spindle (reviewed in [2]).

### 1.3. Other Regulators of Mitotic Spindle Assembly

Apart from microtubule nucleation, MT-associated proteins (MAPs) are essential for spindle assembly. More than 200 MAPs have been described to participate in microtubule nucleation, stabilization, crosslinking and stable kinetochore–microtubule attachments [2,60]. Through these functions, MAPs prevent errors in chromosome alignment and segregation during mitosis [60]. MAPs that regulate microtubules dynamics can be classified into stabilizers and destabilizers. Stabilizers promote microtubule growth, whereas destabilizers increase microtubule catastrophe [61,62]. The α/β-tubulin heterodimers undergo post-translation modifications (PTMs), including acetylation, polyglycylation, polyglutamilation, and tyrosination [63,64,65,66]. Most PTMs occur after microtubule polymerization and their biological roles remain incompletely understood. One exception is phosphorylation of β-tubulin by Cdk1 in mitosis. Anne Fourest-Lieuvin et al. showed that Cdk1 phosphorylates soluble β-tubulin at Serine 172 (S172) (Table 1), thereby impairing its incorporation into growing microtubules in mitotic cells [67].

## 2. Role of Chk1 in DNA Damage Response

**Figure 2:**
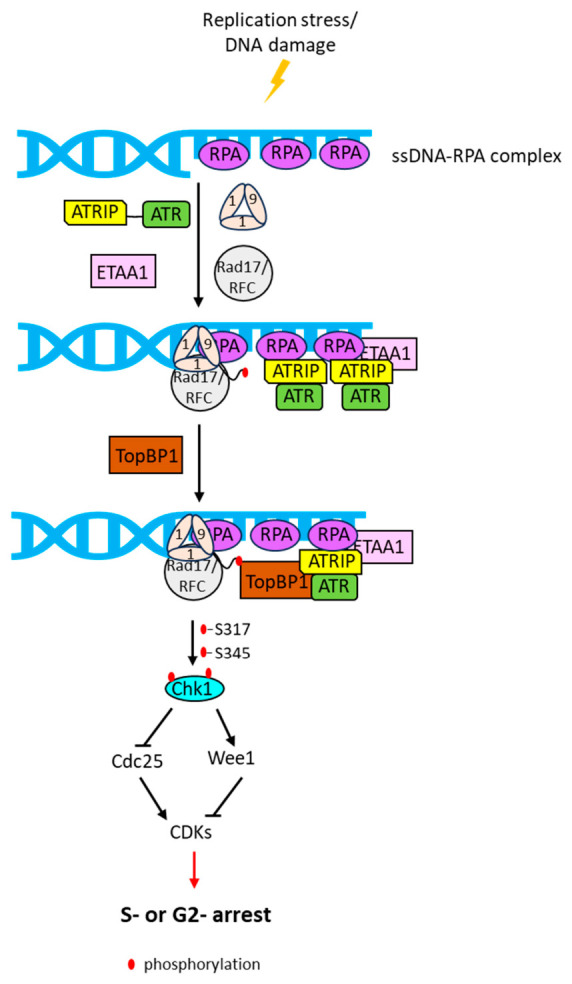
Replication stress or DNA damage response. Upon replication stress or DNA damage, ssDNA is coated by RPA. The RPA-ssDNA complex interacts with the ATRIP-ATR complex, the Rad17-RFC complex and ETAA1. Rad17 recruits the 9-1-1 complex which is recognized by the ATR activator protein, TopBP1, whereas ETAA1 activates ATR in a TopBP1-independent manner. Then, active ATR phosphorylates Chk1 at Serine 317 (S317) and Serine 345 (S345) to fully activate it. Active Chk1 inhibits the family of Cdc25 phosphatases and activates Wee1 kinase, regulating CDKs activity and promoting cell cycle arrest.

Errors in chromosome segregation during mitosis can lead to generation of DNA damage [68]. Checkpoint kinase 1 (Chk1), a Serine/Threonine kinase, is a major regulator of DNA damage response. Under conditions of replication stress, for example in the presence of DNA lesions after UV light and chemical mutagens or absence of crucial replication factors, ATR-Chk1 signaling pathway is activated [69]. In these cases, single-stranded DNA (ssDNA) is formed and becomes coated by replication protein A (RPA) [70,71]. Thereafter, RPA associates with ATR-interacting protein (ATRIP), leading to ataxia-telangiectasia mutated- and Rad3-related (ATR) recruitment to the RPA-ssDNA complex [72]. In addition, the Rad17–replication factor C (RFC) complex interacts with the RPA-ssDNA complex and recruits the Rad9-Rad1-Hus1 (9-1-1) complex [73]. Then, phosphorylated Rad9 subunit of 9-1-1 complex is recognized by BRCT domains I and II of DNA topoisomerase II-binding protein 1 (TopBP1) [74,75]. Through its ATR Activating Domain (AAD), TopBP1 binds to ATRIP-ATR complex and activates ATR [76,77]. Moreover, in mammals, ETAA1 binds directly to RPA and, through its AAD domain, interacts with and activates ATR in a TopBP1-independent manner [78] (Figure 2).

Upon activation, ATR phosphorylates Chk1 at Serine 317 (S317) and Serine 345 (S345) (Table 1) within the C-terminal domain [79,80]. Active Chk1 phosphorylates and inhibits the Cdc25 family of phosphatases and promotes the activation of Wee1 kinase, thereby regulating cyclin-dependent kinase (CDK) activity to promote S- and G2-phase arrest [80,81,82,83] (Figure 2). In the presence of other ssDNA structures, such as dsDNA-ssDNA junctions, Nbs1 subunit of MRN complex recognizes RPA-ssDNA and recruits TopBP1 to activate ATR signaling [84,85].

## 3. Role of Chk1 in Chromosome Segregation

**Figure 3:**
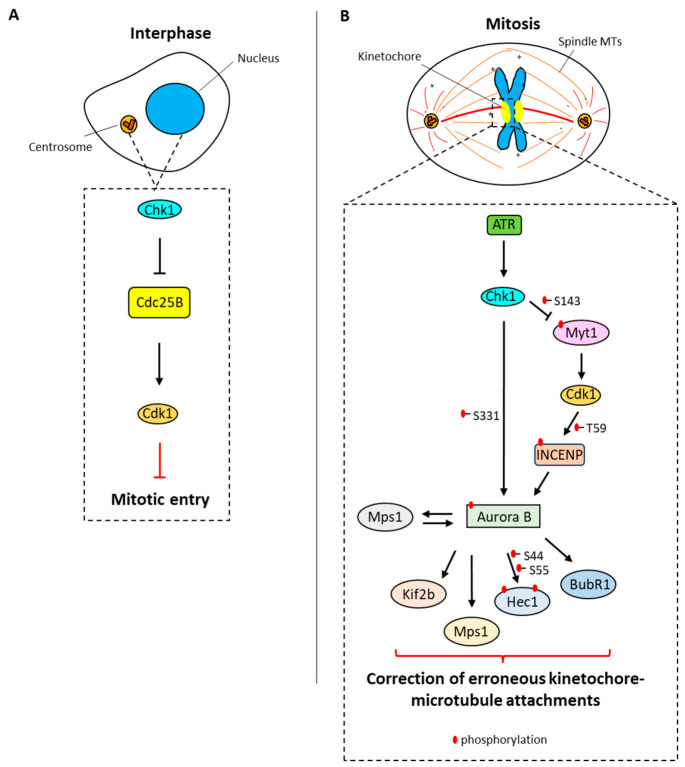
Chk1 is required for mitotic entry and faithful chromosome segregation. (**A**) In the absence of DNA damage in interphase, Chk1 blocks premature mitotic entry by preventing Cdc25B and Cdk1 activation. (**B**) During mitosis, centromeric R-loops promote ATR activation. ATR activates Chk1, which promotes Aurora B activation directly, through its phosphorylation at Serine 331 (S331), or indirectly, through Myt1 phosphorylation at Serine 143 (S143). Active Aurora B recruits Mps1 and BubR1 to kinetochores in the presence of microtubule–kinetochore misattachment to prevent mitotic exit. To correct merotelic microtubule–kinetochore attachments, Chk1 cooperates with Mps1 to regulate MCAK, Kif2b and Hec1 in an Aurora B-S331-dependent manner.

In the absence of DNA damage, human Chk1 localizes at centrosomes and nucleus until the onset of mitosis. Chk1 prevents premature Cdk1 activation by cytoplasmic Cell Division Cycle 25B (Cdc25B) and ensures proper mitotic entry timing [86,87]. During interphase, centrosomal Chk1 phosphorylates Cdc25B at Serine 230 (S230) and at Serine 563 (S563) (Table 1), and it acts as a negative regulator of Cdc25B [88]. Chk1 also localizes to the nucleus, where it suppresses premature activation of Cdk1-Cyclin B [87]. At late G2 phase, Aurora A phosphorylates Cdc25B at Serine 353 (S353) at centrosomes (Table 1), promoting its activation [89,90]. Then, Cdc25B phosphatase promotes activation of Cdk1-Cyclin B complex. Thereafter, Cdk1-Cyclin B complex translocates from the centrosomes to the nucleus, where Cdk1 phosphorylates Chk1 at Serine 286 (S286) and at Serine 301 (S301) (Table 1), promoting Chk1 cytoplasmic release and sustained Cdk1 activity, thereby inducing mitotic entry [87] (Figure 3A).

Also, depletion of Chk1 increases chromosome misalignment, lagging chromosomes and localization of Bub1-related protein 1 (BubR1) to kinetochores during mitosis [91,92]. Furthermore, in the presence of R-loops at centromeres during prometaphase, ATR is activated, Chk1 localizes to kinetochores and phosphorylates Aurora B at Serine 331 (S331) (Table 1), which is required for complete Aurora B activation [93,94]. Aurora B is the catalytic subunit of the Chromosomal Passenger Complex (CPC) also containing INCENP, Survivin and Borealin [95]. INCENP is a scaffold protein of the CPC complex that associates with Borealin and Survivin through its N-terminus to form a three-helix bundle and binds to Aurora B through its C-terminal IN-box [96,97,98]. Upon Aurora B-INCENP binding, Aurora B phosphorylates the IN-box of INCENP on a Thr-Ser-Ser (TSS) motif, while Aurora B also undergoes auto-phosphorylation at Threonine 232 (T232) within its kinase domain, leading to its full activation [99,100,101]. The N-terminus of INCENP is essential for the proper localization of the CPC complex during cell division. At the onset of mitosis, CPC complex localizes to centromeres, whereas on anaphase onset, INCENP binds to Mklp2, promoting CPC relocalization to the spindle midzone and subsequently to the midbody during cytokinesis [102,103,104,105]. In the presence of microtubule–kinetochore attachment defects, active Aurora B promotes BubR1 and Mps1 recruitment to kinetochores hence preventing mitotic exit [92,93,106]. Chk1 also phosphorylates Myt1 kinase at Serine 143 (S143) (Table 1) inhibiting its activity and consequently promoting Cdk1 activation [107]. In turn, Cdk1 could regulate Aurora B localization and activity by phosphorylating INCENP at several residues including Threonine 59 (T59) [107,108] (Figure 3B).

When a single kinetochore is attached with microtubules extending from both spindle poles simultaneously, known as a merotelic attachments, vertebrate cells prevent chromosome missegregation in an Aurora B-mediated manner [109,110,111,112]. Unfortunately, merotelic attachments are common in early mitosis and are not detected by mitotic spindle checkpoint. Their persistence until anaphase onset leads to the formation of lagging chromosomes, promoting chromosomal instability and tumorigenesis [109,113]. Petsalaki et al. showed that active Chk1 phosphorylates Aurora B at Serine 331 (S331) prevents merotelic attachment and lagging chromosomes on the mitotic spindle. This phosphorylation sustains localization of microtubule destabilizers, Kif2b and MCAK, to kinetochores or centromeres, stabilizes the Mps1 binding to kinetochores and is required for phosphorylation of Hec1 at Serine 44 (S44) and at Serine 55 (S55) in metaphase. Also, they showed that inhibition of Mps1 impairs recruitment of Kif2b and MCAK to kinetochores or centromeres, prevents phosphorylation of Hec1 and further increases the percentage of merotelic attachment and lagging chromosomes in Chk1-deficient cells [114]. Furthermore, Mps1 phosphorylates Borealin to regulate Aurora B activity [115] (Figure 3B). These results show that Chk1 promotes faithful chromosomal segregation by enforcing the SAC and by correcting merotelic kinetochore attachments, through Aurora B.

In the absence of tension, as in syntelic or monotelic attachment, the inner centromere-localized Aurora B phosphorylates components of the KMN network at the outer kinetochore, reducing their affinity for MTs and promoting MT-kinetochore reattachment until stable kinetochore–microtubule attachment is re-established [24,33,116,117,118,119,120,121]. Regulation of Aurora B activity has been proposed to involve Chk1 and CPC components, including INCENP and Borealin [25].

## 4. Roles of Chk1 and Chk2 in Cytokinesis

During cytokinesis, Chk1 localizes to the midbody and is phosphorylated at Serine 137 (S137) [92,122]. Peddibhotla et al. showed that abrogation of Chk1 through single-cell antibody microinjection promotes furrow regression, multinucleation, Aurora B mislocalization at the midbody and genomic instability in mammalian cells [123]. Furthermore, Chk1 phosphorylates the O-linked β-N-acetylglucosamine (O-GlcNAc) transferase (OGT) at Serine 20 (S20), promoting OGT stabilization and localization at the midbody. The Chk1-OGT pathway reduces intermediate filament accumulation at the cytoplasmic canal, thereby promoting faithful cytokinesis [124].

Moreover, Chk1 kinase has a role in cytokinesis with chromatin bridges. Chromatin bridges are strands of incompletely segregated chromatin that connect the anaphase poles or daughter nuclei in cytokinesis and have been implicated in tumorigenesis [125,126,127]. Cells harboring chromatin bridge during cytokinesis, delay abscission, the final cut of the intercellular canal, and form structures of polymerized actin, called actin patches, at the bases of the intercellular canal to prevent chromatin breakage [128]. In mammals, this abscission delay is called the “abscission checkpoint” and is dependent on the Aurora B activity [128,129]. Chk1 phosphorylates the Bloom’s syndrome protein helicase (BLM) at Serine 502 (S502) (Table 1) and this phosphorylation stabilizes BLM protein. Depletion of Chk1 or BLM by siRNAs increases the formation of anaphase bridges. However, expression of constitutively phosphorylated BLM-S502D reduces the number of anaphase bridges in Chk1-deficient cells. These results suggest that Chk1 prevents formation of chromatin bridges by phosphorylating BLM and inhibiting its degradation [130].

**Figure 4:**
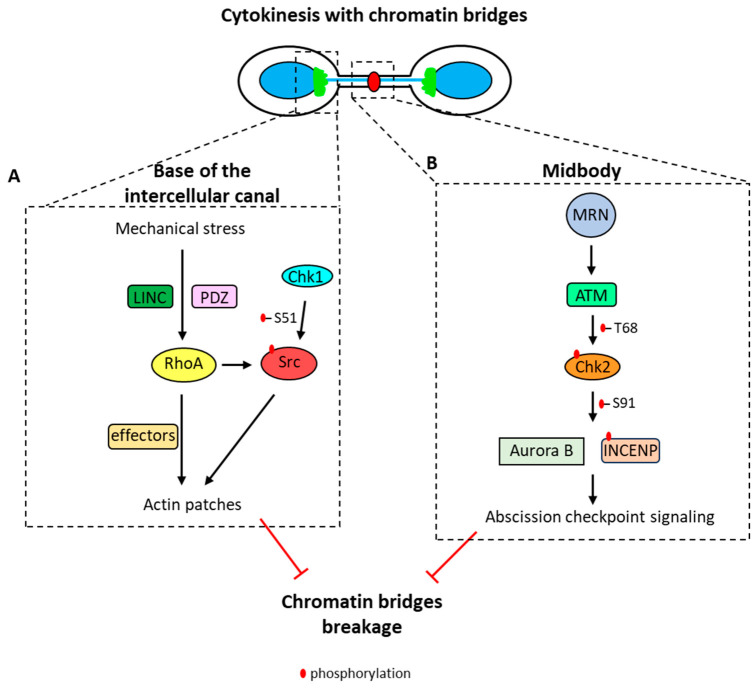
Chk1 and Chk2 prevent chromatin bridge breakage during cytokinesis. (**A**) Nuclear tension activates the LINC-PDZ-RhoA signaling pathway to promote actin patch formation at the bases of the intercellular canal. Chk1 phosphorylates Src at Serine 51 (S51), which functions downstream of RhoA in actin patch formation chromatin bridge stabilization. (**B**) At the midbody, the MRN complex activates ATM. In turn, ATM phosphorylates Chk2 at Threonine 68 (T68) and activate it. Active Chk2 phosphorylates INCENP at Serine 91 (S91) and promotes the localization of the CPC complex to the Flemming body, thereby activating abscission checkpoint and preventing chromatin bridge breakage.

Dandoulaki et al. showed that Src, a nonreceptor tyrosine kinase, localizes to actin patches and is phosphorylated by Chk1 at Serine 51 (S51) to induce full activation of Src kinase. A non-phosphorylatable mutant of Serine 51 to Alanine reduces Src catalytic activity and impairs actin patch formation. These results show a new role of Chk1 in the formation of actin patches and genome stability during cytokinesis [131]. A recent study showed that, in cytokinesis with chromatin bridges, traction forces exerted on daughter nuclei by intact DNA bridges generate nuclear tension. Cells convert this mechanical signal into biochemical signaling, through tension-induced activation of a LINC–PDZ RhoGEF–RhoA signaling pathway, thereby promoting actin patch formation and stabilizing chromatin bridges. RhoA acts upstream of Src signaling in actin patch formation [132] (Figure 4A).

Chk2, a paralogue of Chk1, is implicated in the activation of abscission checkpoint. In cytokinesis with chromatin bridges, the MRN complex localizes to the midbody and is required for ATM activation. Active ATM phosphorylates Chk2 at Threonine 68 (T68) (Table 1), thereby activating Chk2. In turn, active Chk2 phosphorylates INCENP at Serine 91 (S91) (Table 1) promoting localization of CPC components INCENP and Aurora B at the midbody. This delay abscission and prevent chromatin bridge breakage [105] (Figure 4B).

## 5. Novel Role of Chk1 in Mitotic Spindle Formation

### 5.1. Chk1 Is Required for Nucleation and Optimal Formation of Spindle MTs

In a recent study, using confocal or time-lapse microscopy to analyze different cell lines in mitosis, Boutakoglou et al. demonstrated that, in normally segregating cells without drug treatment, Chk1 is required for optimal formation of mitotic spindle during vertebrate cell division by phosphorylating β-tubulin [133]. The researchers showed that inhibition of Chk1, although it does not completely impair mitotic spindle formation, results in diminished spindle microtubule density during prometaphase in diverse vertebrate cell lines. They indicated that the reduction of mitotic spindle density is not caused by early entry into mitosis or DNA damage response, but Chk1 promotes spindle MT density specifically during mitosis in an Aurora B or Cdk1 independent manner. Also, using a microtubule cold recovery assay, the authors showed that inhibition of Chk1 in prometaphase reduces the re-growth capacity of microtubules [133]. These results, together with the localization of active Chk1 at centrosomes from prophase to metaphase, suggest a role for Chk1 in spindle microtubule nucleation and formation.

### 5.2. Chk1 Phosphorylates β-Tubulin at Centrosomes in Prometaphase and Metaphase

Boutakoglou et al. demonstrated that Chk1 interacts with β-tubulin in cell extracts and phosphorylates β-tubulin in vitro. Liquid chromatography–mass spectrometry analysis presented four novel and conserved phosphorylation sites on β-tubulin by Chk1, Threonine 274, Serine 278, Threonine 285 and Threonine 290 [133]. Thereafter, the authors investigated the biological role of these phosphorylation sites in cells and showed that the expression of a non-phosphorylatable β-tubulin mutated at 285 to Alanine (T285A) construct, resistant to β-tubulin siRNA, impairs robust spindle MT density in Chk1 or β-tubulin deficient cells. On the contrary, expression of phosphomimetic β-tubulin mutated at 285 to Glutamic Acid (T285E) rescues the mitotic spindle density despite depletion of Chk1 or β-tubulin [133]. These results suggest that phosphorylation of β-tubulin at Threonine 285 is necessary for optimal spindle microtubule formation.

To further investigate the phosphorylation of β-tubulin at Threonine 285 in mitosis, a phospho-specific antibody (anti-phospho β1-tubulin T285) for the human protein sequence was raised. Impaired localization of phosphorylated β-tubulin at Threonine 285 at the centrosomes, after depletion of Chk1 or β-tubulin, reveals that Chk1 phosphorylates β-tubulin at T285 at centrosomes in prometaphase and metaphase [133].

### 5.3. Role of Phosphorylation of β-Tubulin at Threonine 285 in the Progression of Cell Division

The authors also investigated the role of β-tubulin phosphorylation at Threonine 285 in spindle microtubule nucleation. Using MT re-growth assays and experiments of in vitro tubulin phosphorylation by Chk1 during microtubule polymerization, they showed that Chk1 phosphorylates tubulin at Threonine 285 to promote MT nucleation. Also, they confirmed that Chk1 directly interacts with microtubules in cell extracts and in vitro [133]. Moreover, they showed that lack of phosphorylation of β-tubulin at Threonine 285 disrupts stable attachment between microtubules and kinetochores, resulting in chromosome misalignment, missegregation and delayed anaphase onset due to activation of the spindle checkpoint in human cells [134,135].

The authors also observed that cells lacking phosphorylation of β-tubulin at Threonine 285 exhibit non-centered mitotic spindles in metaphase and produce daughter cells with uneven sizes in cytokinesis. This is interesting because they showed that phosphorylation of β-tubulin at Threonine 285 is implicated in effective cell proliferation [133]. These results suggest that Chk1 phosphorylation at Threonine 285 on β-tubulin promotes spindle formation, thereby ensuring accurate cell proliferation and division.

### 5.4. Upstream Effectors of Chk1 at Centrosomes

To investigate Chk1 activation at mitotic centrosomes, Boutakoglou et al. focused on the implication of ATR, ATRIP and TopBP1 in spindle formation, which are known to activate Chk1 in response to DNA damage. First, they showed that ATR, ATRIP and TopBP1 localize to centrosomes in prometaphase, indicating a direct role of these proteins in mitosis. Second, depletion of ATR, ATRIP and TopBP1 reduced the relative intensity of active Chk1 (pS345) and phosphorylation of β-tubulin at Threonine 285 at centrosomes and impaired the formation of robust spindle microtubules. However, expression of the phosphomimetic β-tubulin-T285E exhibited optimal spindle microtubule density [133]. These results suggest that ATR, ATRIP and TopBP1 are responsible for Chk1 activation at centrosomes during mitosis.

With siRNA-mediated depletion of ATR, ATRIP or TopBP1, the authors showed that ATRIP recruits ATR and TopBP1 to centrosomes in prometaphase. To verify whether the interaction among ATR, ATRIP and TopBP1 is required for Chk1 activation, they engineered two ATRIP variants: the truncated ATRIP del (Δ658–690) and the mutant ATRIP top (LLSS 332 to AAAA), which are incapable of binding to ATR and TopBP1, respectively [76,136]. They found that ATRIP interacts with ATR and TopBP1 to activate Chk1 at mitotic centrosomes leading to phosphorylation of β-tubulin at T285 and promoting optimal polymerization of mitotic spindle microtubules [133].

## 6. Conclusions

**Figure 5:**
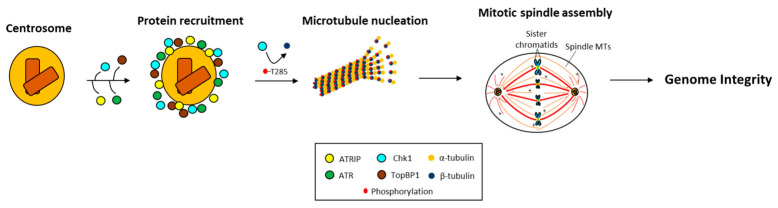
Role of Chk1 in mitotic spindle formation and genome stability. In prometaphase and metaphase, ATRIP localizes at centrosomes and associates with ATR and TopBP1. ATR phosphorylates and activates Chk1, which phosphorylates β-tubulin at Threonine 285 (T285) to promote optimal mitotic spindle formation. Through this mechanism, ATR-Chk1 signaling preserves genome integrity.

The findings of Boutakoglou et al. describe a new role of Chk1 in mitotic spindle formation during cell division [133]. They show that, during mitosis, ATRIP localizes to mitotic centrosomes and promotes the recruitment of ATR and TopBP1. The association of ATRIP with ATR and TopBP1 is required for Chk1 phosphorylation (pS345) and activation by ATR. Thereafter, Chk1 phosphorylates β-tubulin at Threonine 285, a conserved residue in vertebrate cells, in prometaphase and metaphase, thereby promoting spindle microtubule nucleation and optimal mitotic spindle formation. This signaling pathway is important for anaphase onset, accurate chromosome alignment and segregation, formation of equal-sized daughter cells and cell proliferation (Figure 5). These results are consistent with emerging studies demonstrating the role of DNA damage response proteins in unperturbed mitosis in order to preserve genome integrity and highlight novel relationships between mitotic cell division and the DDR [56].

## 7. Open Questions

### 7.1. How Is ATR Recruited to Centrosomes During Mitosis?

The novel role of ATRIP, ATR, TopBP1 and Chk1 in normal mitotic spindle formation raises several questions. First, the mechanism underlying the ATRIP-ATR complex recruitment to centrosomes in prometaphase and metaphase remains to be further investigated. Could ATRIP be recruited to spindle poles by factors such as ETAA1 or Rad17, which are known to promote ATR-Chk1 signaling in response to DNA damage [137]? Furthermore, the interaction of ATR and ATRIP with kinases that localize to centrosomes, such as NIMA-related kinase 1 (Nek1), could activate ATR-Chk1 signaling in the absence of DNA damage [138].

The phosphorylation of β-tubulin at Threonine 285 by Chk1 is the first described post-translational modification of tubulin, which promotes microtubule polymerization and regulates mitotic spindle maturation, contributing to generation of genomically identical daughter cells. Errors in signaling pathways that ensure accurate chromosome segregation and cell proliferation can lead to genetic disorders and birth defects in humans [139,140,141,142]. So, ATR and Chk1 are suggested to function protectively against human diseases and tumorigenesis through their newly identified role in mitotic spindle assembly.

### 7.2. How Does Phosphorylation of β-Tubulin T285 Promote Optimal Microtubule Spindle Density?

**Figure 6:**
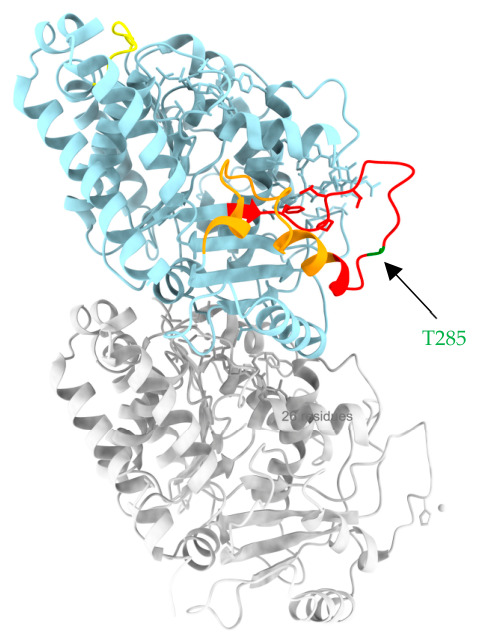
Structural model of the αβ-tubulin dimer highlighting critical assembly elements. The hetero-dimer consists of α-tubulin (light grey) and β-tubulin (light blue). The M-loop (microtubule loop) of β-tubulin, spanning residues 270–290, is highlighted in red. The critical Threonine residue at position 285 (T285), located within the M-loop, is depicted as green spheres to illustrate its spatial positioning. Adjacent structural motifs involved in microtubule stability are also resolved: β-strand S3 (residues 96–103) is colored yellow, and helix H9 (residues 291–310) is colored orange. Molecular graphics were generated using UCSF ChimeraX based on the high-resolution coordinates of PDB ID: 1JFF.

M-loops are essential parts of α-/β-tubulins, which are located between B7 strand and H9 helix and are involved in lateral interaction between protofilaments [143,144]. In β-tubulin, the N terminal end of the M-loop is also important for binding with taxol and taxol-like compounds [8]. Threonine 285 of β-tubulin is located within the M-loop (Figure 6). Perhaps phosphorylation of β-tubulin at Threonine 285 enhances interactions between neighboring protofilaments and increases curvature tolerance promoting microtubule polymerization [145]. Also, T285 phosphorylation of β-tubulin could accommodate interactions between microtubules with MAPs and motor proteins, promoting microtubule assembly [146]. It remains unknown whether another kinase phosphorylates α-tubulin within its M-loop during mitosis, cooperating with β-tubulin phosphorylation at Threonine 285 to promote mitotic spindle formation [9,143].

It is interesting that phosphorylated β-tubulin at T285 localizes to and around centrosomes only during prometaphase and metaphase. One possible explanation is that, following β-tubulin incorporation to the microtubule lattice, the phospho-specific antibody cannot approach the M-loop because of polymerized microtubule conformational changes [147]. In contrast, both wild-type and phosphomimetic β-tubulin-T285E localized along the mitotic spindle. Furthermore, increased levels of γ-tubulin are observed at centrosomes during prometaphase, compared with interphase [52]. At the onset of mitosis, γ-tubulin promotes microtubule nucleation by recruiting α/β-tubulin heterodimers and accommodating their lateral interactions [148]. So, phosphorylation of β-tubulin at Threonine 285 by Chk1 could be required for γ-tubulin-mediated microtubule nucleation in mitosis.

### 7.3. Phosphorylation of β-Tubulin at T285 as a Target for Cancer Therapy

Microtubules are a key target of anticancer therapy. Nevertheless, their implication in many normal cellular functions limits the efficiency of microtubule targeting agents (MTAs) in clinical applications [149]. Researchers have focused on novel agents that are less toxic to non-cancerous cells and overcome chemotherapy resistance in cancer cells [150]. Preclinical trials have demonstrated decreased cancer cell survival by treatment with drugs that simultaneously disrupt microtubule dynamics and inhibit kinases-dependent oncogenic pathways [151,152]. In recent years, new therapeutic agents, with significant potential, have been developed to target the already well-characterized MTA-binding sites (e.g., taxane binding site) and act as microtubule stabilizers or destabilizers [150,153].

A better understanding of microtubule assembly pathways and their roles in cellular processes could contribute to development of novel anticancer drugs with improved effects towards normal cells. Phosphorylation of β-tubulin at Threonine 285 by Chk1 impairs cancer cell proliferation. Because DNA damage response proteins, such as Chk1 or ATR, are implicated in cell survival and proliferation, they represent crucial targets for cancer therapy [154,155,156,157]. It would be interesting to investigate whether impaired tubulin phosphorylation after Chk1 inhibition sensitizes tumor cells to antiproliferative effect of MTA, such as taxanes and alkaloids.

ATR, Chk1 and β-tubulin-T285 are conserved from yeast to higher eukaryotes, raising the possibility that the ATR-β-tubulin signaling axis is evolutionary conserved across different cell types and organisms [133]. Because T285 phosphorylation is important for cell physiology in vertebrate cell lines, dysregulated β-tubulin-T285 phosphorylation may contribute to human genetic disorders and cancer [133]. For example, it will be interesting to examine whether β-tubulin-T285 is hyper-phosphorylated in human cancers, or whether inhibition of T285 phosphorylation has potential therapeutic implications by selectively inhibiting proliferation of cancer cells.

## Figures and Tables

**Table 1:** Summary of kinases, their substrates and phosphorylation sites discussed in this review.

Kinase	Substrate	Phosphorylation Site
ATM	Chk2	Threonine 68
ATR	Chk1	Serine 317, Serine 345
Aurora A	Cdc25B	Serine 353
Aurora B	Hec1	Serine 44, Serine 55
Aurora B	INCENP	Thr-Ser-Ser (TSS) motif
Aurora B	Auto-phosphorylation	Threonine 232
Cdk1	β-tubulin	Serine 172
Cdk1	Chk1	Serine 286, Serine 301
Cdk1	Aurora B	Threonine 59
Chk1	β-tubulin	Threonine 285
Chk1	Cdc25B	Serine 230, Serine 563
Chk1	Aurora B	Serine 331
Chk1	Myt1	Serine 143
Chk1	Src	Serine 51
Chk1	OGT	Serine 20
Chk1	BLM	Serine 502
Chk2	INCENP	Serine 91

## Data Availability

No new data were created or analyzed in this study. Data sharing is not applicable to this article.

## Abbreviations

The following abbreviations are used in this manuscript:

9-1-1	Rad9-Rad1-Hus1
APC/C	Anaphase-promoting complex/cyclosome
ATM	Ataxia-telangiectasia mutated
ATR	Ataxia-telangiectasia mutated- and Rad3-related
ATRIP	ATR-interacting protein
Aurora A	Aurora kinase A
Aurora B	Aurora kinase B
BLM	Bloom’s syndrome protein helicase
BRCA1	Breast cancer type 1 susceptibility protein
Bub1	Budding Uninhibited by Benzimidazoles 1
Bub3	Budding Uninhibited by Benzimidazoles 3
BubR1	BUB1-related protein 1
CCAN	Constitutive Centromere-Associated Network
Cdc20	Cell division cycle protein 20 homolog
Cdc25B	Cell Division Cycle 25B
Cdk1	Cyclin-dependent kinase 1
CENP-A	Centromere protein A
CENP-B	Centromere protein B
CENP-C	Centromere protein C
CENP-E	Centromere protein E
CENP-F	Centromere protein F
Chk1	Checkpoint kinase 1
Chk2	Checkpoint kinase 2
CPC	Chromosomal Passenger Complex
DDR	DNA Damage Response
ETAA1	Ewing tumor-associated antigen 1
GCPs	γ-tubulin complex proteins
Hec1	Highly expressed in cancer 1
Hus1	Hydroxyurea sensitive 1
INCENP	Inner Centromere Protein
K-fibers	Kinetochore fibers
Kif2b	Kinesin family member 2B
KMN	KNL1/Mis12 complex/Ndc80 complex
LINC	Linker of nucleoskeleton and cytoskeleton
Mad1	Mitotic arrest deficient 1
Mad2	Mitotic arrest deficient 2
MAPs	MT-associated proteins
MCAK	Mitotic centromere-associated kinesin
MCC	mitotic checkpoint complex
Mklp2	Mitotic Kinesin-like protein 2
Mps1	Monopolar spindle 1
Mre11	Meiotic recombination 11
MRN	Mre11-Rad50-Nbs1
MTAs	Microtubule targeting agents
MTs	Microtubules
Myt1	Membrane-associated tyrosine- and Threonine-specific cdc2-inhibitory kinase
Nbs1	Nibrin
Nek1	NIMA-related kinase 1
OGT	O-linked β-N-acetylglucosamine (O-GlcNAc) transferase
PDZ RhoGEF	Rho guanine nucleotide exchange factor 11
Plk1	Polo-like kinase 1
PTMs	Post-translational modifications
Rad1	Radiation sensitive 1
Rad17	Radiation sensitive 17
Rad50	Radiation sensitive 50
Rad9	Radiation sensitive 9
RFC	Replication factor C
RhoA	Ras homology family member A
RPA	Replication protein A
SAC	Spindle assembly checkpoint
ssDNA	Single-stranded DNA
TopBP1	DNA topoisomerase II-binding protein 1
TSS	Thr-Ser-Ser
Wee1	Wee1-like protein kinase
γTuRC	γ-tubulin ring complex
γTuSC	γ-tubulin small complex
